# Development of a Shuttle Vector That Transforms at High Frequency for the Emerging Human Fungal Pathogen: *Candida auris*

**DOI:** 10.3390/jof10070477

**Published:** 2024-07-11

**Authors:** Brenden Determann, Jianmin Fu, Brian L. Wickes

**Affiliations:** The Department of Microbiology, Immunology, and Molecular Genetics, The University of Texas Health Science Center at San Antonio, San Antonio, TX 78229-3900, USAfuj@uthscsa.edu (J.F.)

**Keywords:** centromere, electroporation, nourseothricin, plasmid

## Abstract

Routine molecular manipulation of any organism is inefficient and difficult without the existence of a plasmid. Although transformation is possible in *C. auris*, no plasmids are available that can serve as cloning or shuttle vectors. *C. auris* centromeres have been well characterized but have not been explored further as molecular tools. We tested *C. auris* centromeric sequences to identify which, if any, could be used to create a plasmid that was stably maintained after transformation. We cloned all seven *C. auris* centromeric sequences and tested them for transformation frequency and stability. Transformation frequency varied significantly; however, one was found to transform at a very high frequency. A 1.7 Kb subclone of this sequence was used to construct a shuttle vector. The vector was stable with selection and maintained at ~1 copy per cell but could be easily lost when selection was removed, which suggested that the properties of the centromeric sequence were more Autonomously Replicating Sequence (ARS)-like than centromere-like when part of a plasmid. Rescue of this plasmid from transformed *C. auris* cells into *E. coli* revealed that it remained intact after the initial *C. auris* transformation, even when carrying large inserts. The plasmid was found to be able to transform all four clades of *C. auris*, with varying frequencies. This plasmid is an important new reagent in the *C. auris* molecular toolbox, which will enhance the investigation of this human fungal pathogen.

## 1. Introduction

In 2009, *Candida auris* was isolated and identified from the external ear canal of a Japanese patient [1]. This newly emerging *Candida* species has become one of the most notable nosocomial fungal pathogens, as it has quickly spread throughout the world [2,3,4]. Interestingly, it has appeared in four different geographical locations, and this has led to the division of this species into distinct clades, which include the East Asia, South Asia, Africa, and South America clades [5,6]. A recent isolate from Iran has been identified and proposed as the fifth clade for this species [7,8]. Individual clades differ from each other by at least 10,000 SNPs, and the newly identified Iranian clade is 200,000 SNPs removed from its next closest clade [8]. The independent appearance of *C. auris* on various continents with no clear evidence of a single ancestor remains curious from an evolutionary perspective [2].

The rapid emergence of *C. auris* and initial poor characterization led to common biochemical misidentification [9]. As most laboratories and commercial vendors began to update their practices and identification systems, the misidentification of *C. auris* strains has decreased [9,10]. However, even with a better diagnosis, mortality rates can range from 30 to 60%, depending on patient status and country [4], and are driven in large part by multidrug resistance [11]. A majority of *C. auris* strains are resistant to one antifungal class, with many demonstrating resistance to two classes, and a small percentage being resistant to all three antifungal drug classes [12]. As nosocomial infections continue to rise, the Centers for Disease Control has labeled *C. auris* a high-priority threat to hospitals and healthcare facilities [13]. The rapid appearance and spread of *C. auris*, its multidrug resistance potential, and high mortality rate have led to intense interest in this fungus.

Unfortunately, as is the case for most human fungal pathogens, the development of a molecular toolbox for *C. auris* has been slow; however, progress is being made. For example, there are multiple CRISPR strategies [14,15,16], transformation by both electroporation and lithium acetate [17,18], multiple selectable and fluorescence markers [14,19,20,21], and almost 200 genome sequences available in public databases, many of which are annotated [6]. While a plasmid-based construct has been used for transformation of *C. auris*, it is integrative, similar to the *S. cerevisiae* YIp plasmids [22], which do not contain sequences that allow autonomous replication. Therefore, the development of an autonomously replicating plasmid that can serve as a shuttle vector would add significantly to the *C. auris* molecular toolbox.

Shuttle vectors facilitate the cloning, complementation, and transformation of host strains and typically lead to a rapid increase in potential molecular manipulations of fungi for which they are developed. However, because of poor sequence conservation, there are few efficient interspecies fungal shuttle vectors, thus necessitating the development of new plasmids from scratch from the organism of interest. For a plasmid to function in a host cell, two important components are required: selectable transformation markers (which exist for *C. auris* [18,19]) and a sequence that allows for autonomous replication once the plasmid is inside the host cell. While elements such as an autonomously replicating sequence (ARS) confer this function, they are hard to identify based on sequence alone. Centromeres, on the other hand, have been found to have conserved sequences and can confer an autonomous replication ability on plasmids [23,24]. This function allows plasmids to be maintained episomally, and if they can be easily partitioned from the transformed cells, it also allows for the recycling of the original host phenotype, such as auxotrophy or drug sensitivity. The presence of bacterial elements on the plasmid (replication origin, ampicillin, or other drug-resistance marker) confers the ability of the plasmid to act as a shuttle vector since it can be maintained in host bacteria as well.

In 2021, a research group was able to biochemically characterize and map seven distinct centromeric sequences from the genome of *C. auris* [25]. Using chromatin immunoprecipitation and comparative ChIP-seq genome analysis, they were able to define these centromeric features and suggested that they played an essential role in how *C. auris* might have emerged as a multidrug-resistant nosocomial pathogen. Interestingly, while related, the centromeres differed significantly at the sequence level. For example, not all the centromeres possessed a core conserved sequence within the defined centromeric region. Within the non-conserved sequences, long stretches of AT bases, with some as long as 40 bp, were observed; however, these regions did not align well. The correlation of ChIP-seq results (seven single peaks) with the number of *C. auris* chromosomes (seven), conserved protein sequence with other CENP-A family members, and conserved localization patterns of centromeres being typically found at the nuclear periphery suggested that these sequences were true *C. auris* centromeres [25]. The centromeric-like (CEN) sequences and their small sizes led us to hypothesize that they could be incorporated into a plasmid with a selectable marker to enable the plasmid to be maintained episomally and function as a shuttle vector in *C. auris*. When we tested this hypothesis, we observed that some, but not all, of the CEN sequences transformed *C. auris* at a high frequency and conferred episomal function on a plasmid, with one of the centromeric sequences found to be much more efficient for transformation than the others. This study is the first report of the development of a functional plasmid for the transformation of *C. auris*.

## 2. Materials and Methods

### 2.1. Strains, Media, and Reagents

*Candida auris* wild-type strain, WSA 3432, was used as the host strain. Other WSA strains are denoted in Table 1. YPD broth consisted of 1% yeast extract, 2% Bacto peptone, and 2% dextrose. YPD agar was prepared from broth via solidification with 2.0% agar. Yeast strains were kept at −80 °C in frozen stocks of YPD broth and 15% glycerol. YPD-NAT was prepared fresh the day of the experiment, and NAT (nourseothricin) concentrations were determined empirically for each clade or strain if needed, due to background growth of untransformed cells, and ranged from 150 to 250 μg/mL. For WSA 3432, which was the main strain used in this study, it was 150 μg/mL in both agar and broth. Luria-Bertani (LB) consisted of 1% tryptone, 0.5% yeast extract, and 1% sodium chloride and was used for growth of bacterial strains. When needed, carbenicillin was added to a final concentration of 100 μg/mL. Bacterial strains were stored frozen at −80 °C in LB broth with 25% glycerol. Super-optimal broth with catabolite repression (SOC) was prepared by adding 2.0 g of tryptone, 0.5 g of yeast extract, 1.0 mL of 1 M NaCl, and 250 μL of 1 M KCl to 95 mL of dH_2_O. The pH was adjusted to 7.0, followed by autoclaving. The medium was cooled to room temperature before 1.0 mL of sterile 2 M MgCl_2_ and 1.0 mL of sterile 2 M dextrose solution were added. Sterile dH_2_O was added until a final volume of 100 mL was reached. *Escherichia coli* One Shot TOP 10 Electrocompetent cells (Invitrogen, Carlsbad, CA, USA) were utilized for all bacterial transformations.

Nourseothricin sulfate (NAT) (Gold Biotechnology, St. Louis, MO, USA) was prepared fresh for each experiment and used for the selection of yeast transformants. DL-Dithiothreitol (DTT), Lithium Acetate, Carbenicillin, TE, Dextrose, NaCl, KCl, MgCl_2,_ glycerol, and Sorbitol were obtained from Sigma-Aldrich (St. Louis, MO, USA). Tryptone, Bacto peptone, and yeast extract were obtained from Fisher (Fisher Scientific, Pittsburgh, PA, USA). PCR primers (Table 2) were designed using MacVector software (MacVector Inc., Apex, NC, USA) and obtained from Eurofins Genomics, Inc. (Louisville, KY, USA). Real-time PCR primers, probes, and mastermix were obtained from Integrated DNA Technologies (IDT, Coralville, IA, USA). Restriction enzymes and ligase were obtained from New England Biolabs (Beverly, MA, USA). KOD Hot Start and KOD Xtreme polymerases were obtained from EMD Millipore Corp. (Burlington, MA, USA) and were used for high-fidelity and/or long-distance PCR reactions.

### 2.2. DNA Extraction and Isolation

Genomic DNA extractions from *C. auris* were conducted by subculturing cells from a −80 °C frozen stock onto fresh YPD agar, growing for 48 h, and then extracting as previously described [26]. Briefly, a single colony was transferred into a 2 mL screw-cap tube (BioSpec Products Inc, Bartlesville, OK, USA) containing 200 μL PrepMan Ultra (Fisher Scientific, Inc.) and 100 μL of 0.5 mm glass beads (BioSpec Products, Inc.). The tubes were placed in a 99 °C heating block for 10 min and bead beat in a BioSpec Mini-beadbeater (BioSpec Products, Inc.) for 30 s. This sample was centrifuged, and the supernatant was transferred to a new 1.5 mL microfuge tube. Aliquots were used directly as template DNA for PCR amplification.

PCR amplicons or restriction digests that needed to be recovered from a gel were purified with the QIAquick Gel Extraction Kit (Qiagen, Hilden, Germany) after electrophoresis. Other PCR reactions that did not need gel purification were cleaned using the QIAquick *PCR Purification* Kit (Qiagen). Qiagen samples were eluted in 20 μL of Buffer EB (10 mM Tris HCl, pH 8.5) or sterile H_2_O, and DNA concentration was determined by NanoDrop 2000 Spectrophotometer (Thermo Scientific, Wilmington, DE, USA).

Plasmids were prepared by inoculating the host strain into 3 mL of LB carbenicillin broth. Tubes were incubated overnight at 37 °C, with shaking at 250 RPM. Plasmids were then isolated using a QIAprep Spin Miniprep Kit (Qiagen). DNA was eluted with 20 μL of TE (10 mM Tris HCl pH 8.5, 1 mM EDTA) and quantified using a NanoDrop 2000 Spectrophotometer or Quantus Fluorometer using QuantiFlour ONE dsDNA Dye (Promega, Inc., Madison, WI, USA).

### 2.3. Plasmid Generation

#### 2.3.1. p1114

The NAT gene in a cassette containing a *Candida albicans* promoter and terminator [27] was recovered from plasmid p624 [28] as a *SmaI* and *SpeI* fragment and ligated into pBluescript-SK(+) (Stratagene, Inc., La Jolla, CA, USA) cut with the same enzymes. The ligation reaction was electroporated into One Shot TOP 10 Electrocompetent *E. coli* cells. After transformation, cells were recovered in SOC media for 1 h at 230 RPM at 37 °C and then spread onto LB carbenicillin agar plates, which were incubated overnight at 37 °C. Transformants were purified, and plasmids were recovered from transformants using the QIAprep Spin Miniprep Kit. Plasmid DNA was digested with *SmaI* and *SpeI* and run on a gel to confirm the presence of the NAT cassette. This plasmid was denoted as p1114 (Table 3).

#### 2.3.2. *C. auris* Centromere Clones

Seven *C. auris* B8441 centromeric clones from the wild-type *C. auris* strain WSA 3432 were recovered using primers with a *NotI* site on one primer and a *SacII* or *SpeI* site on the other primer. The plasmids were labelled p1120, p1122, p1123, p1124, p1129, p1130, and p1134 (Table 3). The predicted amplicon regions were determined by using the centromere sequences previously identified to search the genome of B8441, which was the source of the original centromere sequences [25]. Primers were designed with MacVector to include the centromere and additional flanking DNA outside the centromere sequence. PCR reactions consisted of 1.0 μL of WSA 3432 genomic DNA, corresponding to 1–10 ng of DNA; 2.5 μL of 10x buffer; 2.0 μL of 25 mM MgSO_4_; 2.5 μL of 2 mM dNTP; 0.75 μL of both forward and reverse primers (10 μM); 0.50 μL of KOD Hot Start polymerase; and nuclease-free water to 25 μL. PCR cycling conditions consisted of 2 min at 95 °C for initial denaturing, 35 cycles of 20 s at 98 °C, 10 s at varying annealing temperatures depending on primer pair, a 3 min extension time at 70 °C, and a final extension of 5 min at 70 °C. Samples were run on a 1% agarose gel, stained by ethidium bromide, and imaged with a Bio-Rad Gel Doc XR+ System (Bio-Rad, Inc., Hercules, CA, USA).

PCR amplicons were cleaned and then digested with *NotI* and *SacII.* Then, 1 μg of digested, cleaned amplicon DNA from each centromere was ligated into p1114 (digested with the same enzymes) overnight at 16 °C, using T4 DNA ligase. After that, 1 μL and a 1:10 dilution of the ligation reaction were electroporated into 30 μL of One Shot TOP 10 Electrocompetent *E. coli* cells in a 1 mm electroporation cuvette (Fisher Scientific) using a BioRad Gene Pulser (BioRad, Inc.) set at 200 Ohms, 2.0 kV, and 25 μF. Samples were recovered in 200 μL of SOC outgrowth medium and incubated for 1 h at 230 RPM and 37 °C. Then, 50 μL and 150 μL of cells were plated onto LB carbenicillin agar and incubated overnight at 37 °C. Plasmids were isolated and digested with *NotI* and *SacII* to confirm the presence of the centromere inserts. The seven centromere plasmids are indicated in Table 3.

#### 2.3.3. p1153

The optimized plasmid that yielded the highest transformation frequency was designated p1153. This plasmid was derived from the primer walk results of p1120, which contained the original full-length CEN 4 locus and yielded plasmids p1137-p1142, as well as p1143, p1146, p1148, p1149, p1151, and p1152 (see below and Table 3). The CEN 4 subclone encompassing the minimal functional region was swapped into p1152 as a 1.7 kb *NotI*-*SacII* fragment. Plasmid p1152 was digested with *NotI* and *SacII*, and the CEN 4 fragment was recovered via gel purification. Plasmid p1120 was amplified by primers CEN4.NotI.6188.F and CEN4.SacII.2963.F, using the PCR recipe for the CEN 4 subclone PCR—except that the extension time on the PCR reaction was shortened to 2 min. The sample was purified with the QIAquick PCR Purification Kit, digested with *NotI* and *SacII*, and cleaned. The fragment was ligated into the p1152 vector backbone and then electroporated into One Shot TOP 10 Electrocompetent *E. coli*. The new plasmid was recovered, confirmed to have the CEN 4 subclone by *NotI* and *SacII* digestion, and denoted p1153 (Table 3). This plasmid was deposited with Addgene (ID# 222930).

#### 2.3.4. p1210, p1213

Two plasmids were prepared to test the carrying capacity of p1153 (Table 3). p1210 was a 11.6 Kb plasmid prepared by cloning a 5 Kb insert from the *ADE1* locus, using CaurisADE1.5Kb.F and CaurisADE1.5Kb.R to amplify a fragment that was ligated into p1153 after the digestion of the fragment and plasmid with *SpeI* and *NotI*. Moreover, p1213 was a 16.6 Kb plasmid prepared by cloning a 10 Kb insert from the *ADE1* locus, using CaurisADE1.10Kb.F and CaurisADE1.10Kb.R to amplify a fragment that was ligated into p1153 after digestion of the fragment and plasmid with *SpeI* and *NotI*.

### 2.4. Transformation of C. auris

Transformation of *C. auris* was performed by electroporation, based on previous studies with modification [29,30]. On Day 1 of the transformation, *C. auris* was streaked onto YPD agar and incubated at 30 °C for two days. On Day 3, a single *C. auris* colony was inoculated into a 15 mL snap-cap tube (Fisher) that contained 3 mL of prewarmed YPD broth and shaken at 200 RPM at 30 °C overnight, with the caps loose. On Day 4, the 3 mL saturated seed culture (5 × 10^8^ CFU) was used to prepare a fresh 100 mL YPD flask culture. The seed culture was diluted 1:1000 in prewarmed YPD broth, and then 1.0 mL of this dilution was added to 100 mL of prewarmed YPD broth in a 250 mL flask. The flask was incubated overnight for 15–18 h at 30 °C and 200 RPM. The following morning, when the cells reached an OD_600_ of 1.8–2.0, 50 mL of the overnight culture was transferred to a 50 mL falcon tube (Fisher) and centrifuged for 5 min at 4500× *g* in an Eppendorf 5804 R Benchtop Centrifuge (Eppendorf, Inc., Enfield, CT, USA). The supernatant was discarded, and the pellet was resuspended in 8 mL of sterile H_2_O, 1 mL of 10× TE (pH 8.0), and 1 mL of 1 M Lithium Acetate (LiOAc) (pH 7.5). The suspension was vortexed to resuspend the pellet and then shaken for 1 h at 200 RPM and 30 °C. After 1 h, 250 μL of 1 M DTT was added, followed by incubation for another 30 min. The cells were then pelleted, washed in 40 mL of sterile H_2_O, and centrifuged for 5 min at 4500× *g*. The supernatant was again discarded, and the cells were resuspended in 25 mL of ice-cold sterile H_2_O and then centrifuged for 5 min at 4500× *g*. The supernatant was discarded, and 5 mL of ice-cold 1 M sorbitol was added to the Falcon tube, which was vortexed and then centrifuged for 5 min at 4500× *g*. The supernatant was discarded, and 50 μL of ice-cold 1 M sorbitol was added to the pellet, which was resuspended using a sterile 1 mL pipet tip. The yield per 50 mL is enough cells to perform approximately 4 electroporations. The remainder was frozen at −70 °C and remained competent for at least 6 months.

To transform *C. auris*, a sterile 1.5 mL microfuge tube with 40 μL of competent yeast cells and 10 ng of plasmid DNA in a volume 10 μL was prepared on ice by gently mixing cells and DNA. The sample was transferred to a 2 mm cuvette (Fisher) on ice and then electroporated using a Bio-Rad Gene Pulser set to 1.8 kV, 200 Ohms, and 25 μF. After electroporation, cells were washed with 1 mL of 1 M sorbitol and transferred to a 1.5 mL sterile microfuge tube, which was centrifuged for 5 min at 1500× *g* in an Eppendorf 5424 microfuge (Eppendorf, Inc.). The supernatant was discarded, and 1 mL of YPD broth was used to resuspend the cells, which were then transferred into a 15 mL snap-cap tube (Fisher) that was shaken for 2 h at 200 RPM and 30 °C with the caps loose. Cells were then plated onto freshly prepared YPD-NAT agar plates (typically in 100 μL aliquots), which were incubated at 30 °C for two-to-four days. Unless otherwise indicated, transformations were performed three times, in triplicate.

### 2.5. Identification of the Minimal Centromeric Region

Plasmid p1120 (CEN 4) was chosen to define the minimal essential centromere region for shuttle vector development based on transformation frequency and plasmid stability. The design of plasmid subclones was performed using MacVector software to develop a primer walking strategy for subclone generation. Primer sites were mapped and designed approximately 500 bp apart from both ends of the original CEN 4 cloned fragment in plasmid p1120 so that there were overlapping clones from both the *NotI* end (p1137, p1138, p1139, p1140, p1141, and p1142) and the *SacII* end (p1143, p1146, p1148, p1149, p1151, and p1152) (Table 3). *NotI* or *SacII*-containing primers in combination with a vector primer were used to amplify p1120 template DNA to yield increasingly smaller subclones from either side of the CEN 4 insert. The PCR reaction, which was a long-distance amplification, consisted of 1 μL of DNA (10–100 pg), 12.5 μL of 2× Buffer, 5.0 μL of 2 mM dNTP, 0.75 μL of both forward and reverse primers (10 μM), 0.5 μL of KOD Xtreme polymerase (EMD Millipore Corp, Billerica MA, USA), and 4.5 μL of nuclease-free water. PCR conditions were 2:20 min at 94° for initial denaturation, 35 cycles of 1 s at 98 °C, 30 s at 60 °C, 6 min extension at 68 °C, and a final extension of 2 min at 68 °C. Subclone amplicons were purified with the QIAquick PCR Purification Kit and then digested with either *NotI* or *SacII*, which flank the centromere cloning site at either end. The *NotI* or *SacII* digestion cuts at the end of the PCR primer and the vector site, allowing for easy recircularization after self-ligation. Digestions were purified and self-ligated using T4 DNA ligase and then electroporated into One Shot TOP 10 Electrocompetent *E. coli*. After transformation, cells were recovered in SOC media for 1 h at 230 RPM (Innova 4000, New Brunswick Scientific, Inc., Edison, NJ, USA) at 37 °C and then spread onto LB carbenicillin agar to recover individual CEN 4 *NotI* or *SacII* subclones. Plasmid DNA was recovered from each subclone and transformed into *C. auris* after confirming subclone size. The minimal centromeric region was identified by determining the transformation frequency of each plasmid subclone and by visualizing colonies that grew on transformation plates using a Leica EC4 digital HD microscope with LAS EZ imaging software 3.4 (Leica Microsystems, Inc., Deerfield, IL, USA).

### 2.6. Determination of Plasmid Fate after Transformation

Using *S. cerevisiae* as a model, plasmids that contain a centromere should be more stable compared to plasmids that have just an ARS sequence or 2-micron circle. CEN sequences ensure that plasmids are partitioned into daughter cells efficiently in single or low copies, potentially mimicking a chromosome. ARS and 2-micron-containing plasmids typically are present in higher copies but do not partition as efficiently into daughter cells, leaving some daughter cells without a plasmid [23]. For plasmids with a CEN sequence, if selection is removed, these plasmids can still be partitioned into daughter cells whereas ARS or 2-micron plasmids can be lost quickly due to mis-segregation.

We used a segregation assay [31] to determine whether the plasmid behaved episomally once it was transformed into *C. auris* or became integrated. Episomal plasmids would not be partitioned correctly, in the absence of selection, to the daughter cell and would eventually be lost, which can be detected by non-growing patches on YPD-NAT agar. Patching was performed by removing approximately 1 × 10^6^ cells from a seed culture with a sterile plastic loop and inoculating a 10 mm circular spot on an agar plate, while taking care not to leave visible clumps of cells in the patch. We tested the CEN sequences with the three highest transformation frequencies (CEN 3, CEN 4, and CEN 7). On Day 1 of the segregation assay, single-colony transformants were purified onto fresh YPD-NAT agar media. On Day 2, isolated colonies were patched onto YPD-NAT agar, which served as the master plate for individual transformants, and YPD agar without nourseothricin (selection removed). On Day 3, patched clones from the YPD plates were transferred to new YPD agar plates. On Day 4, clones from the YPD plate were patched back onto YPD-NAT agar to score for plasmid loss, which was identified by non-growing patches. On Day 5, growing and non-growing patches were counted to determine the percentage of clones that did not grow after selection was removed, which would represent those transformants that lost the plasmid due to the absence of nourseothricin selection.

Because we were interested in developing a stable shuttle vector that could replicate in *C. auris* and *E. coli* and could be rescued from *C. auris* into *E. coli* via transformation with *C. auris* DNA containing the plasmid, we used plasmid rescue [32] to determine if *C. auris* plasmids could be recovered intact and unrearranged in *E. coli* after transformation into *C. auris*. Isolated clones from a *C. auris* transformation with p1153 were inoculated into 3 mL of YPD-NAT broth and incubated for 18 h at 200 RPM and 30 °C. The culture was pelleted and washed in 500 μL of sterile H_2_O and resuspended in the same volume of sterile water. Plasmids were extracted with the QIAprep Spin Miniprep Kit (Qiagen). DNA was eluted from the column with 30 μL of TE (10 mM Tris HCl pH 8.5, 1 mM EDTA) or sterile H_2_O and then measured using a NanoDrop 2000 Spectrophotometer. Then, 1.0 μL total DNA was electroporated into One Shot TOP 10 Electrocompetent *E. coli*. After transformation, cells were recovered in SOC media for 1 h at 230 RPM and at 37 °C, as described above, and then plated onto LB carbenicillin agar plates, which were incubated overnight at 37 °C. Plasmid isolation was performed on purified colonies, followed by digestion with *EcoRI* or *XbaI*. Digests were run on a 1% agarose gel to confirm the presence of an intact rescued plasmid by comparison to the restriction enzyme digestion pattern of p1153 cut with the same enzyme.

### 2.7. qPCR Copy-Number Determination of Plasmids

qPCR was used to determine the copy number of the plasmid. Two sequences were targeted; actin was used as a control because the copy number is one in the *C. auris* genome [33], and the NAT gene was chosen since it is not present in the *C. auris* genome but present as the selectable marker on the plasmid. FAM (5′ 6-FAM; Fluorescein) (IDT) was used for the *C. auris* actin gene fluorophore, and Cy5 (Cyanine-5) (IDT) was used as the NAT gene fluorophore during RT-PCR.

Seven large and seven small colonies that grew on YPD-NAT agar after transformation with p1153 were selected for qPCR. A buffer composed of 10 mM sodium phosphate, 1.2 M sorbitol solution, and 5.0 mg/mL of Lyticase from *Arthrobacter luteus* (Sigma-Aldrich, St. Louis, MO, USA) was added to spheroplast cells. Both colony types were incubated initially for 30 min at 37 °C to degrade the fungal cell wall. The reaction was then placed in a thermocycler for 15 min at 94 °C, then −80 °C for 5 min, and 94° again for 15 min for two complete incubation rounds to lyse the spheroplasts. The sample was chilled for 10 min on ice before being centrifuged. The supernatant was transferred to a new microfuge tube, and DNA was measured by a Quantus Fluorometer using QuantiFlour ONE dsDNA Dye (Promega, Madison, WI, USA).

qPCR primers (C.aur.ACT.qPCR.F, C.aur.ACT.qPCR.R, and C.aur.NAT.qPCR.F, C.aur.NAT.qPCR.F) were diluted to 10 μM, and probes were diluted to 4 μM concentrations. The sample mixture contained 5 μL of Master Mix (IDT), 0.5 μL of both forward and reverse primers, 0.5 μL of gene-specific probe, 1.0 μL of genomic DNA (5 ng), and 2.5 μL of nuclease-free water. Samples were run in triplicate using a BioRad CFX 96 Real-Time PCR Detection System (BioRad), according to the manufacturer’s fast-cycling protocol: 95 °C polymerase activation for 3 min (1×), 95 °C for 5 s, annealing and extension at 60 °C for 30 s, for a total of 40 cycles. The formula 2^−(∆∆)CT^ was utilized to obtain the copy number of individual colonies from both small- and large-colony samples. The experiment was repeated five times for both colony types. A two-sample *t*-test was performed on qPCR data and evaluated for significance (*p* < 0.05) of copy number between colony types.

### 2.8. Plasmid Stability

We next determined the fate of the plasmid in the absence of selection. To test these characteristics, WSA 3432 transformed with p1153 was patched onto YPD agar with or without NAT, which served as the seed cultures for non-selective and selective conditions, respectively, and incubated for 4 days at 30 °C. Suspensions were then prepared and adjusted by hemocytometer count to 1 × 10^8^ CFU/mL in dH_2_O. Serial dilutions were made, and suspensions were plated onto YPD and YPD-NAT agar plates, which were incubated at 30 °C for 2 days and counted.

We also performed a variation of this experiment using broth culture. Immediately after, electroporation cells were recovered, as in our normal electroporation protocol (wash with 1 mL of 1 M sorbitol and resuspended in 1 mL of YPD broth). A 100 μL aliquot of this suspension was plated onto YPD-NAT agar for a T_0_ time point. The remaining 900 μL was incubated as per normal electroporation conditions, by shaking the tube at 30 °C at 200 RPM—except that the incubation period was extended for three days. Subsequent platings onto YPD-NAT plates occurred at 2 h, 24 h, 48 h, and 72 h. These plates were incubated at 30 °C for 2 days and counted. Data were analyzed by *t*-test and considered significant at *p* < 0.05. Experiments were performed three times, in triplicate.

## 3. Results

### 3.1. C. auris CEN Shuttle Vectors Generate a High Transformation Frequency, Which Is CEN-Clone Specific

**Figure 1:**
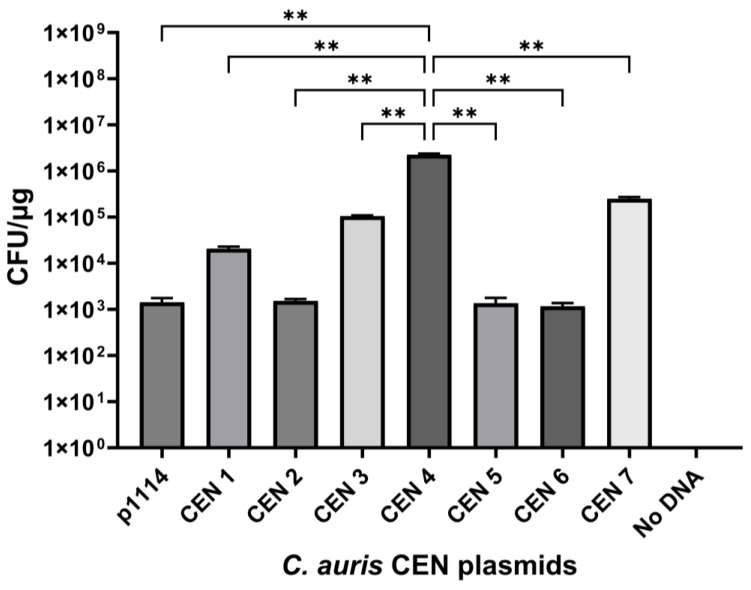
Individual *C. auris* centromere transformation frequency. Centromere fragments amplified by PCR were cloned into p1114 and transformed into WSA 3432. Transformants were plated onto YPD-NAT plates in serial dilution and then counted. The number of transformants was expressed as CFU per μg of DNA and was determined using three biological replicates and three technical replicates. Plasmids containing CEN 3, 4, and 7 showed the highest transformation frequencies, with CEN 4 yielding significantly more transformants than all other CEN sequences. Significance was determined by ANOVA with Tukey’s test. Differences were considered significant at *p* < 0.05. Significance level was ** = *p* < 0.01.

Individual CEN amplicons were ligated into p1114 using enzymes *NotI* and *SacII* (CEN 6 was ligated with *SpeI-NotI* instead of *SacII-NotI)*. Centromeric shuttle vector whole-plasmid sizes are denoted in Table 3. A total of 100 ng of plasmid DNA from p1114 and each CEN plasmid was transformed into *C. auris*, and plates were counted for CFU and then normalized. Transformant numbers varied from 1.2 × 10^3^ to 2.3 × 10^6^. Plasmids p1120 (CEN 4), p1122 (CEN 7), and p1130 (CEN 3) produced the largest number of transformants in *C. auris*; however, CEN 4 produced almost 10× the number of transformants than the next-highest centromere (Figure 1).

**Figure 2:**
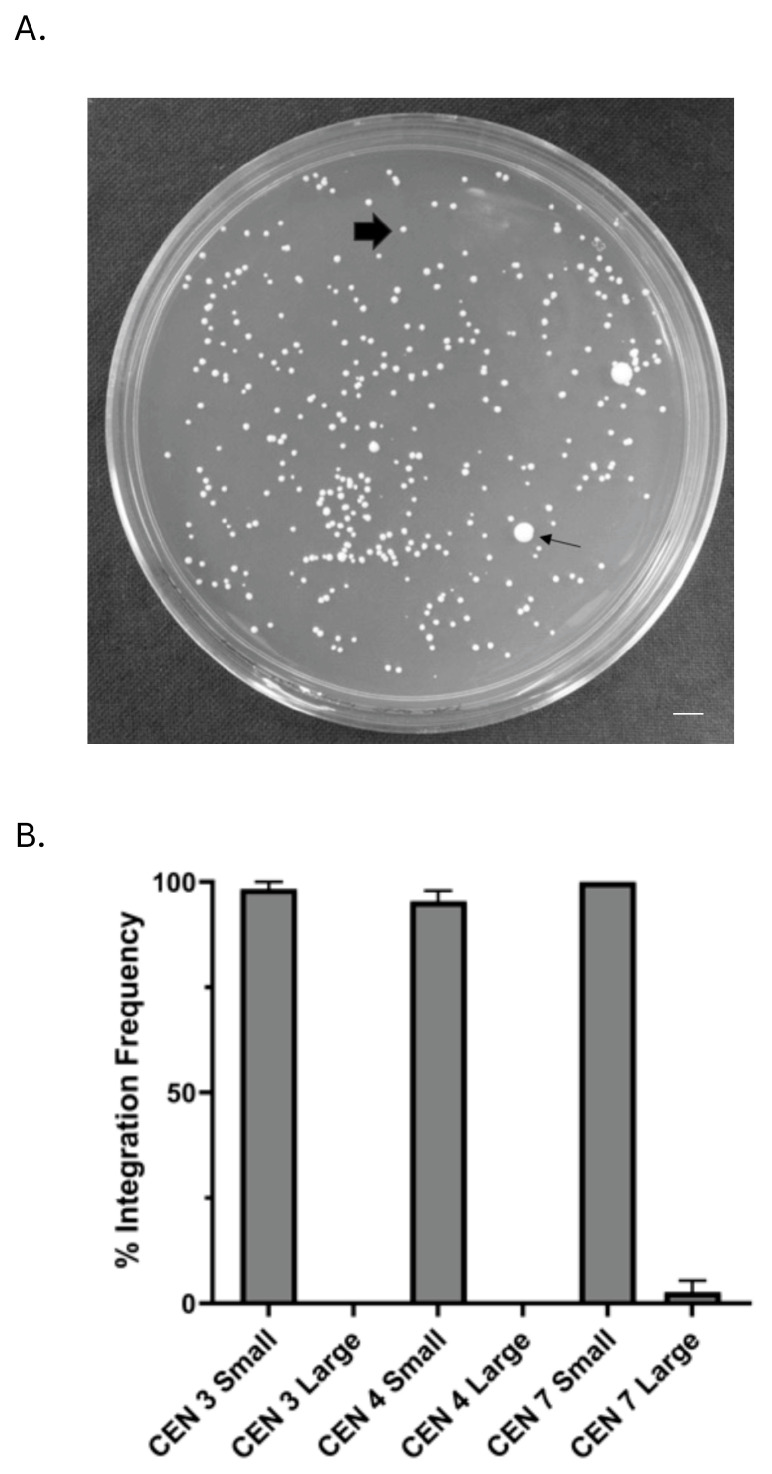
Discrimination of large- and small-colony transformants. (**A**) Centromeric plasmids yielded two colony types after transformation; large colonies (small arrowhead) that were 5–10× bigger than smaller colonies (large, filled arrowhead). Bar = 5 mm. (**B**) In order to characterize large and small colonies, a segregation assay was performed on purified large and small colonies from transformation plates. The results showed that, regardless of CEN source, almost 100% of the small colonies lost the plasmid in the absence of NAT selection, while very few of the large colonies lost the plasmid in the absence of selection, suggesting that small colonies contained episomal plasmids, whereas large colonies contained integrated plasmids.

Transformation resulted in two colony types, large and small (Figure 2A). Moreover, p1114, which contained only the NAT marker and no CEN sequence, produced only large colonies. Plasmids containing CEN 3, CEN 4, and CEN 7 produced the largest number of both colony sizes, which were evaluated to determine the nature of these plasmids using a segregation test. Large colonies, which were the minority of the total transformants, rarely segregated the nourseothricin-resistant phenotype, suggesting plasmid integration, whereas small colonies rapidly lost the resistant phenotype at very high frequencies, suggesting episomal plasmids (Figure 2B).

### 3.2. CEN 4 Insert Truncation by Primer Walking

**Figure 3:**
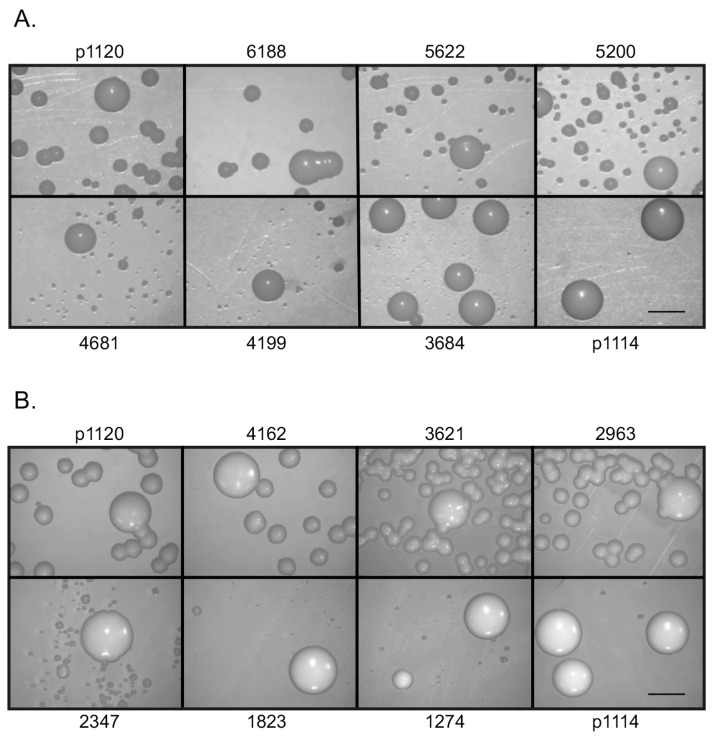
Determination of the minimal functional region of CEN 4. (**A**) Transformation results for subclones that resulted from *NotI* digestion after PCR with primers containing a *NotI* site (Table 2), followed by religation and transformation of purified plasmids from *E. coli* plasmid preparations. Transformation with plasmid subclones containing increasingly smaller CEN 4 regions from p1120 revealed that while large colonies (which are integrative transformants) remain the same size, after NotI subclone 6188, small colonies become increasingly smaller as the CEN 4 region is reduced. Subclone 3684 small colonies are barely distinguishable from p1114, which contains the NAT marker only. Bar = 3 mm. (**B**) Transformation results for subclones that resulted from *SacII* digestion, religation, and transformation, as above. The *SacII* subclones were derived from the opposite site of CEN 4 and showed small colony size decreasing after the 2963 subclone. Plates were photographed at 2.5×, with an exposure time of 110 ms. Bar = 3 mm.

**Figure 4:**
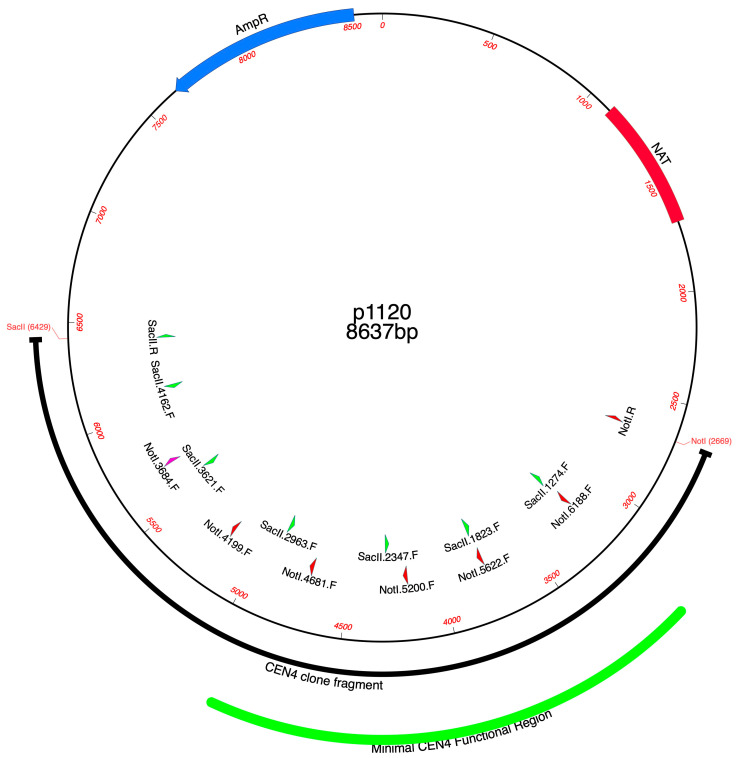
Map of the minimal functional region of CEN 4. Map of p1120 showing the original CEN 4 PCR insert (thick black line) and the minimal functional region (thick green outer line). This region was delineated by Not I.6188.F and SacII.2963.F.

Because plasmid p1120 (CEN 4) exhibited the greatest transformation frequency, it was investigated further as a potential shuttle vector. Shuttle vectors need to be as small as possible to facilitate the cloning of potentially large fragments. Therefore, we mapped the CEN 4 insert by primer walk at approximately 500 bp intervals to determine how much of the original flanking DNA could be deleted while still retaining episomal plasmid function. *NotI* subclones that contained fragments delineated by primer Not I.5200.F and larger (Not I.5622.F and Not I.6188.F) all yielded > 1 × 10^5^ transformants/μg (1.3 × 10^5^–1.64 × 10^5^). However, fragments smaller than Not I.6188.F began to show the smaller, episomal plasmid colonies decreasing in size (Figure 3A). Therefore, we selected Not I.6188.F as one CEN 4 subclone boundary. Similar results were seen for the Sac II subclones of CEN 4. S*acII-*subcloned plasmids also eventually produced colonies that decreased substantially in size when fragments smaller than the Sac II 2963.F were tested. Fragments Sac II.2963-SacII 4162 all yielded > 7 × 10^4^ transformants/μg (7.75 × 10^4^–1.50 × 10^5^). However, fragments smaller than SacII 2963.F produced smaller episomal plasmid colonies that decreased abruptly in size. These results suggested that the SacII.2963.F site was the other boundary of the CEN 4 minimal functional region (Figure 3B and Figure 4). An examination of the sequence bounded by these sites revealed that two long poly T stretches, 17 bp and 29 bp in length, lie within the Not I.6188.F and Sac II 2963.F boundary of the CEN 4 subclone (Supplementary Figure S1).

### 3.3. Shuttle Vector Transformation Efficiency and Optimization

**Figure 5:**
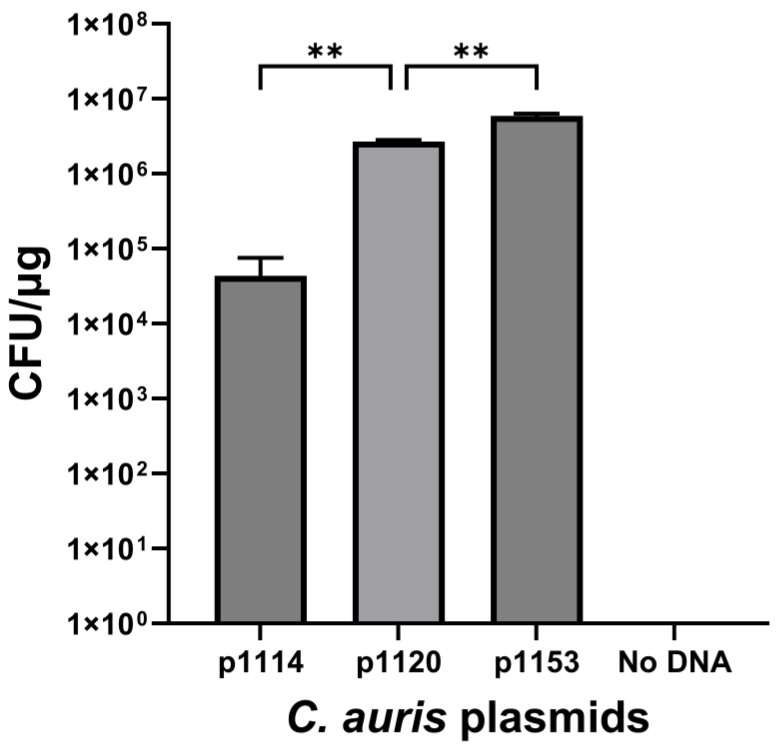
Transformation frequencies of p1114, p1120, and p1153. The minimal functional region of the CEN 4 clone was used to create p1153, the *C. auris* centromeric plasmid containing a 1.7 Kb subclone of the CEN 4 region. This plasmid was compared for transformation frequency to the initial CEN 4 clone (p1120) and the NAT1 plasmid without a centromeric sequence (p1114). Plasmid p1153 yielded significantly more transformants (*p* < 0.05) than p1120, which contained the original CEN 4 subclone, and p1114, which did not contain a CEN 4 subclone. Results were from three independent experiments performed in triplicate. Data were analyzed by ANOVA. Significance level was ** = *p* < 0.01.

The shortest fully functional centromeric fragment comprising the region spanning the NotI.6188.F and SacII.2963.F primers was used to create p1153. This centromeric fragment was reduced to 1.7 kb from the original 3.6 kb CEN 4 clone. To confirm that the shortened CEN 4 fragment still retained the function of the original clone, plasmids p1114, p1120, and p1153 were all transformed under the same conditions to determine transformation frequencies. Respectively, p1120, the original centromere clone, yielded almost 100× more transformants than p1114, which was the NAT resistance plasmid without a centromere. Plasmid p1153 yielded more than twice as many transformants as p1120 (Figure 5).

**Figure 6:**
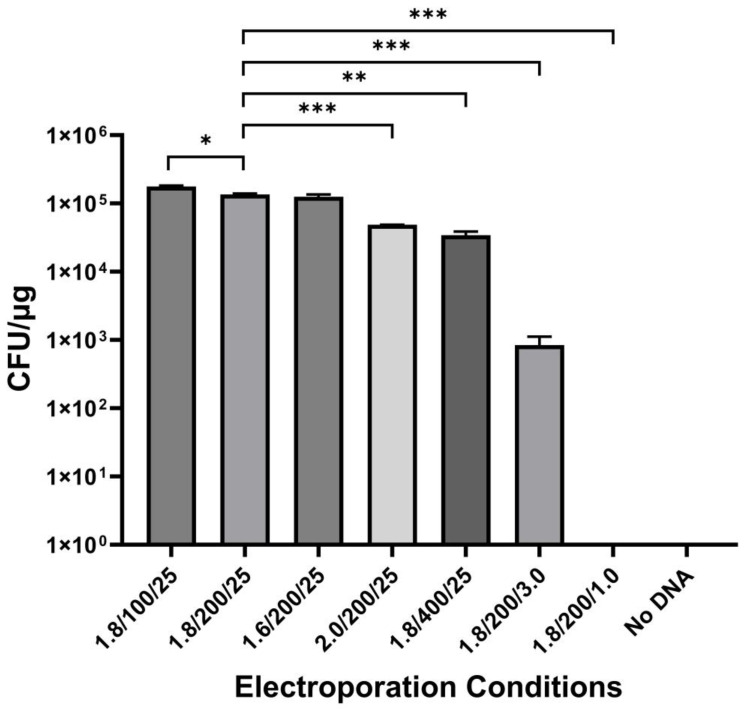
Optimization of electroporation conditions. Various electroporation parameters were modified to find the conditions that yielded the most transformants. The standard electroporation conditions (1.8 kV, 200 Ohms, and 25 μF) yielded significantly more transformants than all other conditions, except 1.6 kV, 200 Ohms, and 25 μF; and 1.8 kV, 100 Ohms, and 25 μF. Electroporation settings of 1.8 kV, 100 Ohms, and 25 μF yielded significantly more transformants than all other settings (*p* < 0.05), including the initial standard electroporation conditions. Experiments were repeated three times, in triplicate, and compared to the standard electroporation conditions of 1.8 kV, 200 Ohms, and 25 μF. Data were analyzed by a paired two-tailed *t*-test with significance set at *p* < 0.05. Significance levels were * = *p* < 0.05, ** = *p* < 0.01, *** = *p* < 0.001.

Lastly, we optimized transformation parameters using p1153, starting with our initial standard settings of 1.8 kV, 200 Ohms, and 25 μF. When the voltage was increased to 2.0 kV, there was a drop in CFU, whereas when the voltage was decreased to 1.6 kV, there was no significant change (Figure 6). When we decreased Ohms to 100, there was an increase in CFU, whereas CFUs decreased when we increased Ohms to 400. Our initial microfarad setting was 25 μF, which was the maximum setting on the Bio-Rad Pulse Controller. When this setting was decreased to 3.0 μF or 1.0 μF, there was a significant drop in both transformations, with 1.0 μF yielding no transformants. We concluded that optimizing the electroporation settings from 1.8 kV, 200 Ohms, and 25 μF to 1.8 kV, 100 Ohms, and 25 μF yielded the highest transformation frequency but noted that a variety of settings yielded large numbers of transformants, which should be useful when optimizing transformation using different electroporators.

### 3.4. Plasmid p1153 Can Be Rescued Intact in E. coli

**Figure 7:**
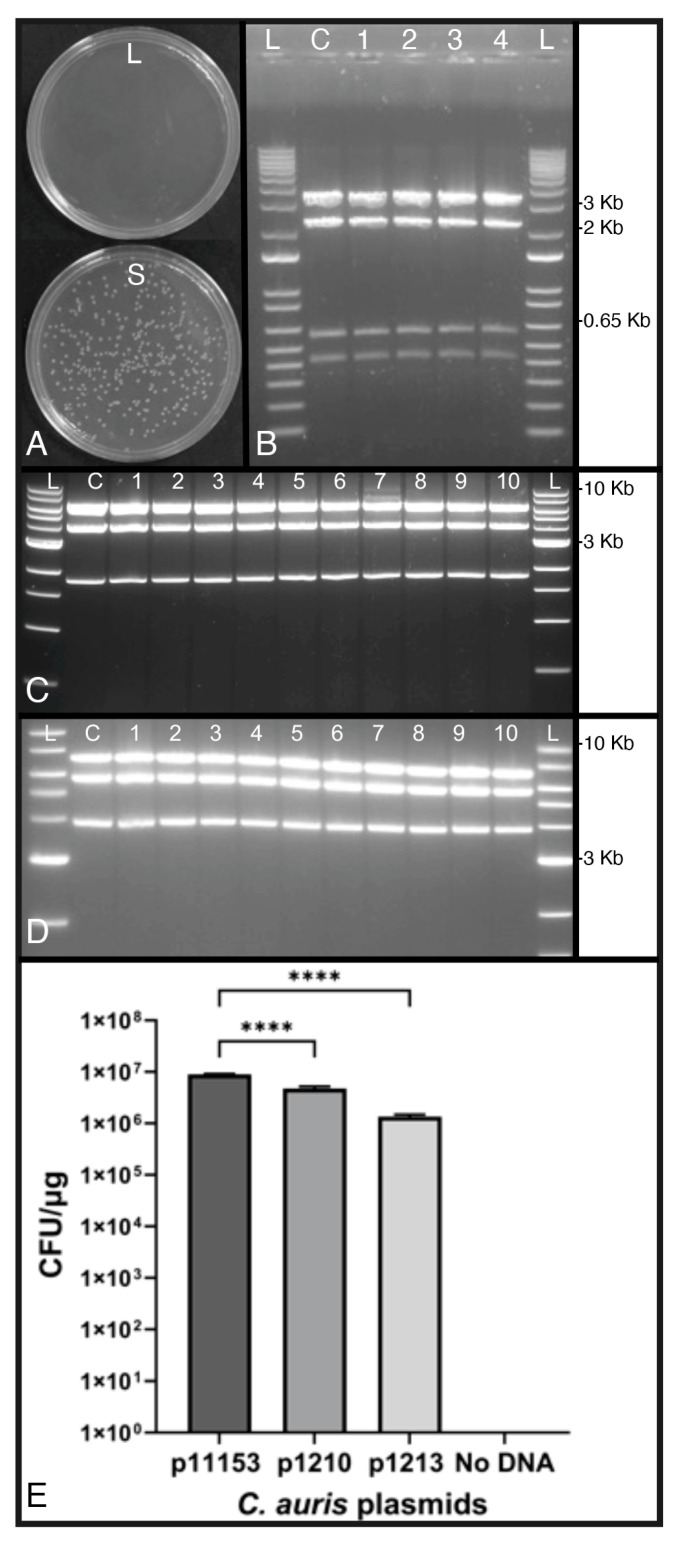
Stability of p1153. (**A**) Transformation of *E. coli* with total DNA prepared from transformed large (L)- and small (S)-colony DNA of *C. auris,* showing that only small-colony total DNA contains episomal plasmids, which are capable of being rescued by transformation of *E. coli*. (**B**) *EcoRI* restriction digest pattern of p1153 (6.6 kb) rescued from *E. coli* after DNA extraction from small-colony *C. auris* transformants. L = ladder, C = control p1153 plasmid, 1–4 plasmids rescued from *E. coli* after transformation with small-colony *C. auris* total DNA. All plasmids show identical bands of 3.4 kb, 2.2 kb, 0.59 kb, and 0.42 kb (11 bp band is not visible). (**C**) *XbaI* digestion of p1210 (11.6 Kb) showing predicted bands of 6.0 kb, 3.9 Kb, and 1.7 Kb. L = ladder, C = control p1210 plasmid, 1–10 plasmids rescued in *E. coli* after transformation with small-colony *C. auris* total DNA. (**D**) *XbaI* digestion of p1213 (16.6 Kb) showing predicted bands of 7.0 kb, 5.7 Kb, and 3.9 Kb. L = ladder, C = control p1210 plasmid, 1–10 plasmids rescued from *E. coli* after transformation with small-colony *C. auris* total DNA. (**E**) Transformation frequencies of p1153, p1210, and p1213, which corresponded to size. p1153 is the smallest plasmid, while p1213 is the largest. The results suggest that plasmid size influences transformation frequency, which would be expected; however, although p1153 yielded significantly more transformants than the two larger plasmids (*p* < 0.05 by ANOVA), the transformation frequency was still good for all three plasmids. Significance level was **** = *p* < 0.0001.

The total DNA prepared from small and large colonies after the transformation of WSA 3432 with p1153 was electroporated into *E. coli*. Large-colony DNA extracts yielded no *E. coli* transformants, while small-colony DNA extracts yielded abundant transformants (Figure 7A). Small-colony-derived plasmids recovered from bacterial transformants digested by *EcoRI* displayed patterns that matched the control plasmid, p1153 (Figure 7B). These results suggested that p1153 was stably maintained as an episomal vector in *C. auris* after transformation. The failure of DNA obtained from large-colony transformants to transform *E. coli* was again consistent with previous observations that large-colony *C. auris* transformants resulted from plasmid integration. To test the carrying capacity of p1153, 5.0 Kb and 10.0 Kb inserts were cloned into p1153. Using the same *E. coli* rescue strategy followed by *XbaI* digestion, as above, p1210 and p1213 both remained unrearranged after transformation into *C. auris* and rescue in *E. coli* (Figure 7C,D and Supplementary Figure S2). When we compared the transformation frequencies of all three plasmids, we noted that the transformation frequency decreased with size; however, p1213, with a 10 Kb insert and total size of >16 Kb, still transformed at a high frequency (Figure 7E). 

### 3.5. NAT1 Copy-Number Determination Using qPCR Analysis

Plasmid copy numbers can vary depending on the regulatory element that they carry for replication. In *S. cerevisiae*, centromere-based plasmids tend to be low-copy plasmids compared to ARS or 2-micron circle replication sequences [34,35]. After the transformation of p1153 into *C. auris*, we determined the plasmid copy numbers for both small and large transformants using qPCR (Supplementary Figure S3). The qPCR results revealed an average copy number of 2.02 for large colonies and 1.29 for small colonies (*p* < 0.05), suggesting that p1153 behaves like a centromere and is indeed low copy. The larger value of the large-colony qPCR may reflect an integration mechanism that results in a duplication of the NAT resistance marker, which would be consistent with the larger size of these colonies due to increased resistance to nourseothricin.

### 3.6. Transformation of p1153 into Various C. auris Clades

Because the *C. auris* species can be separated into multiple clades, we evaluated the ability of plasmid p1153 to transform strains of the different *C. auris* clades. Fungal strains were transformed with 10 ng of plasmid DNA with varying nourseothricin concentrations, which were determined empirically to completely suppress background growth. The clade I strains WSA 3426 and WSA 3533 both required 200 μg/mL of nourseothricin, while WSA 3432 and WSA 3534 required 150 μg/mL of nourseothricin. The clade II strain WSA 3425 required 200 μg/mL of nourseothricin, and the clade III strain WSA 3427, required 200 μg/mL of nourseothricin. The clade IV strain 3535 required 200 μg/mL of NAT. WSA 3543 (clade III) and WSA 3528 (clade III) both required 250 μg/mL nourseothricin.

**Figure 8:**
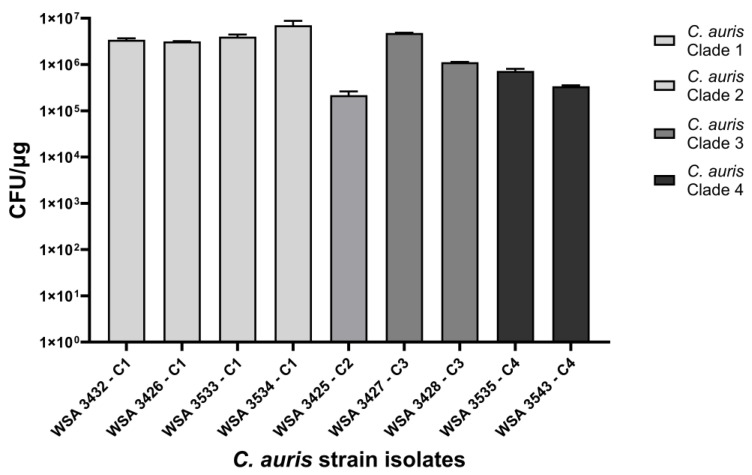
Transformation of *C. auris* clades I–IV with p1153. In general, clade 1 isolates, which were the source of CEN 4, transformed at higher frequencies than the other clades. However, enough samples were not available to rank clade-transformation frequencies, including access to a clade 5 [7].

Transformation frequencies ranged from 2.2 × 10^5^ to 7 × 10^6^ CFU/μg. Clade 1 had two strains that produced more transformants than the original clade I strain, WSA 3432 (Figure 8). However, other members of clade I transformed well, while clades II-IV varied in transformation frequency. These results suggest that p1153 may be able to function as a shuttle vector for the species *C. auris*, regardless of clade. However, we suspect that, depending on strain and clade, additional optimization may be needed, particularly with regard to the nourseothricin concentration.

### 3.7. Plasmid Maintenance with and without NAT Selection

**Figure 9:**
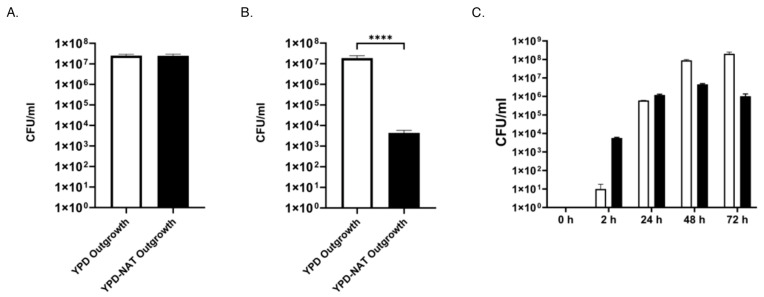
The effect of seed culture growth on agar with or without NAT selection on plasmid loss. (**A**) Seed culture grown under selective conditions on YPD-NAT prior to plating onto YPD and YPD-NAT. The white column represents CFUs that appear on YPD agar (YPD Outgrowth) from the seed culture grown on YPD-NAT. The black column represents CFUs that appear after plating the same seed culture suspension onto YPD-NAT agar (YPD-NAT Outgrowth). There was no significant difference between the CFUs that appeared on YPD agar (2.51 × 10^7^) and the number of NAT resistant colonies that appeared after plating onto YPD-NAT agar (2.47 × 10^7^) after outgrowth. (**B**) Seed culture grown under non-selective conditions on YPD (without NAT) prior to plating onto YPD agar (YPD Outgrowth) and YPD-NAT agar (YPD-NAT Outgrowth). The white column represents CFUs that appear on YPD agar without NAT (YPD Outgrowth). The black column represents CFUs that appear on YPD-NAT (YPD-NAT Outgrowth) after plating cells from the same seed culture suspension. The difference between the YPD Outgrowth CFUs (1.88 × 10^7^) and the NAT-resistant CFUs (4.37 × 10^3^) that grew on YPD-NAT agar (YPD-NAT Outgrowth) was significant (*p* < 3.54 × 10^−11^), as the number of NAT^+^ colonies was reduced by almost 4 orders of magnitude. (**C**) Broth culture under non-selective conditions leads to plasmid loss. Immediately after electroporation, transformants were rescued into YPD broth without NAT and plated onto YPD-NAT agar at various time points. Episomal transformants (black columns) initially predominated, while integrative transformants (white columns) were present at low levels at the earliest time point (2 h). With time, integrative transformants eventually become the majority after 3 days in broth culture, as episomal transformants gradually lose the plasmid. The two transformant types could be distinguished by plating onto YPD-NAT agar and letting these plates grow for two days, until large colonies (integrative transformants) and small colonies (episomal transformants) could be identified. Significance level was **** = *p* < 0.0001.

Because it is essential to distinguish plasmid fate after transformation, we tested transformation outcomes with and without NAT selection and made additional observations concerning the large colonies that appear as a minority population on primary transformation plates. Figure 9A shows that if selection is maintained in the seed culture by including NAT in the YPD agar, cells retain the plasmid after plating onto YPD-NAT since we observed no significant difference between the CFUs of cells that were grown under NAT during seed culture and then plated onto YPD and YPD-NAT. However, Figure 9B shows that if NAT selection is not present during the seed culture, cells will lose the plasmid. Growing cells as a seed culture on YPD only and then plating onto YPD agar and YPD-NAT agar revealed a significant reduction in plasmid-containing CFUs on YPD-NAT. Figure 9C shows the outcome of integrative and episomal transformants after prolonged incubation under non-selective conditions. Integrative transformants, in the form of large colonies, are a tiny minority of initial transformants but appear as soon as two hours after transformation, at the time that episomal transformants appear. However, in the absence of NAT selection, episomal transformants eventually become a minority of the population, as these cells lose the plasmid. In this experiment we did not transfer cells into fresh media so it is likely that, with additional subculturing, integrated transformants would quickly overtake the episomal population until episomal cells could no longer be detected.

## 4. Discussion

In this study, a centromere sequence-based shuttle vector was developed, and transformation conditions were optimized to successfully expand the limited molecular toolbox for *Candida auris*. As nosocomial infections continue to rise, this shuttle vector will serve as a gateway to numerous new ways to manipulate *C. auris* at the molecular and genetic level. Although the clades of *C. auris* can be separated by large differences in excess of 200,000 SNPs [8], it is noteworthy that this vector was able to transform all clades. However, there will likely be additional optimization steps required for each specific strain, as we found differences in nourseothricin resistance depending on the strain, and differences in transformation frequency. Electroporation conditions may also need to be optimized depending on the strain, and certainly if the electroporator is different from the model we used. Surprisingly, although we tested all seven centromere sequences for transformation ability, frequencies varied extensively. This variation may be due to the centromere structure in *C. auris*, which varies somewhat from the *S. cerevisiae* model. Narayanan et al. initially observed that *C. auris* lacks pericentric heterochromatin [25,36]. This domain flanks centromeres and plays an essential role in faithful chromosomal segregation [37], which may explain the observation of rapid loss of the plasmid when selection is removed. With none of the *C. auris* centromeres possessing a core sequence and instead being unique in all seven regions, these differences potentially contributed to the functional differences we observed in the transformation frequency of each CEN sequence.

We did not attempt to test centromeres from other clades; however, it may be more efficient for investigators that work with a preferred strain from a different clade to clone their own clade-specific centromere, although it is probably unlikely that the primers we used in this study would amplify CEN sequences from other clades, particularly distant clades. In this case, if there is enough homology using the original B8441 CEN sequences to identify corresponding CEN sequences from the genome sequences of strains from other clades, the same strategy we used for cloning the different CEN sequences should be applicable for cloning CEN sequences from other clades. This strategy was straightforward, as we designed primers based on the genome sequence of the centromere region, included additional non-centromere flanking sequence, and then added a restriction site on each primer that allowed easy cloning into our nourseothricin resistance vector. Importantly, if this strategy is used, it would be important to keep in mind that only three of the seven CEN sequences transformed with high efficiency, and CEN 4 was significantly better than the other two CEN sequences. Therefore, it may be necessary to test and subclone each centromeric sequence from a preferred strain if p1153 is not suitable. We found a wide range of transformation efficiencies depending on the CEN subclone; however, it is unclear why the transformation efficiency varies with the cloned CEN sequence. Although CEN 4 was the only centromere we examined functionally, an investigation of the sequence revealed the presence of two long poly (T) stretches within the subclone. Smaller CEN 4 subclones that did not contain these regions lost functionality and either yielded no transformants or very small colonies. Lorenz and Papon noted that poly (T) stretches are found in or near all *C. auris* centromeric regions, so it is likely that these sequences play crucial roles in centromeric function [38].

There are very few fungal models of centromere function, with *S. cerevisiae* perhaps being the best described. Unfortunately, while *S. cerevisiae* molecular methods have provided a useful road map for human fungal pathogen research, the efficiency of a given molecular tool in fungal pathogens almost always is far less than the *S. cerevisiae* model. Outside of *C. glabrata* and now *C. auris*, none of the major human fungal pathogens has an efficient shuttle vector, although we note that a *C. glabrata* vector is also a CEN/ARS vector, which uses a NAT marker [39]. Therefore, trying to gauge centromere function in *C. auris* using *S. cerevisiae* as the standard may be unrealistic. For example, when grown in the absence of selection, the *C. auris* CEN plasmid was lost quickly. This observation has been made in *S. cerevisiae* under some circumstances as well [40]; however, the *C. auris* CEN sequence seems to be less stable than the *S. cerevisiae* equivalent and more prone to partitioning errors. Additionally, while centromere sequences confer plasmid stability, variation can occur. In *S. cerevisiae*, for example, the CEN plasmid copy number can vary, with some cells carrying multiple plasmids and other cells losing the plasmid [41]. Although we cloned the predicted centromere region and substantial flanking DNA, it is possible that full centromere function, as defined in *S. cerevisiae* centromeric plasmids, may require additional flanking DNA beyond what we cloned with each centromere locus. Alternatively, centromeric function may require a much larger piece of DNA in the vector, or the CEN sequence may not function effectively on a circular molecule. We did not attempt to define individual centromere and ARS regions, but it is possible that our clones may have truncated sequences required for maximum stability. In fact, Clark and Carbon previously showed that both CEN and ARS regions are required for maximum plasmid stability [42]. The *C. auris* plasmid appears to have some, but not all, of the characteristics described for the *S. cerevisiae* CEN plasmid. Our plasmid transformed at high frequency, was present in low copies, and could carry large inserts. However, in contrast to *S. cerevisiae* CEN plasmids, it was unstable in the absence of selection.

Importantly, our goal for this study was not to develop a vector that behaved as a predicted centromere plasmid would; instead, we wanted to develop a plasmid that transformed at high frequency, did not rearrange, and could be segregated from transformed cells easily. Because our plasmid is lost quickly in the absence of selection, true centromeric-like function of the CEN 4 sequence may be minimal or possibly defective. However, the rapid loss of the plasmid, once selection is removed, is fortuitous because selectable markers are usually limited for fungal pathogens due to the reliance on dominant markers that do not require auxotrophies. When fungal pathogens have auxotrophies, this phenotype can reduce virulence or can bias other studies that require a wild-type genome. Dominant drug-resistant markers are typically not part of the native genome and do not require a corresponding mutant as a transformation host. Therefore, easy loss of the plasmid quickly recycles the nourseothricin-sensitive phenotype, which will allow the plasmid to be used in another transformation round. For the small percentage of the transformants that were identified as integrative, they could be distinguished by a larger colony phenotype. While the integration of an episomal plasmid is an undesirable outcome, for the *C. auris* plasmid, these events can easily be distinguished based on the much larger size of integrated plasmids. However, if a transformation suspension is cultured in broth and not plated onto agar, large-colony overgrowth could be a concern.

Although expense may limit access to the instrumentation required to transform *C. auris* by electroporation, the transformation frequency was generally quite high, which is essential for some plasmid-based molecular manipulations. Electroporation also seems to be robust, as we recovered transformants over a variety of conditions and transformed large plasmids with high frequency. Because these conditions depend on the settings available with a specific electroporator model, transformation with a different electroporator may not be possible with the exact settings that we used. Therefore, having some variation in electroporator settings that still yields transformants will allow other electroporator models to be used that do not match ours. Furthermore, the rescue of p1153 and the increasingly larger p1210 and p1213 plasmids from *E. coli* showed no evidence of rearrangement, which demonstrates that this transformation system results in stable transformants at a high frequency. We also found that cells remained competent at −70 °C for ≥6 months and consistently transformed at a high frequency.

In summary, we developed the first autonomously replicating plasmid for *C. auris* that transforms at a high frequency, replicates faithfully, and can be easily segregated to recycle the selectable marker. This reagent will become a part of the *C. auris* molecular toolbox and will enable the use of inducible promoters, library construction, mutant complementation, heterologous gene expression, and a variety of other manipulations that require a shuttle vector.

## Figures and Tables

**Table 1:** *Candida auris* strains.

Strain	Clade	Origin	Contributor, and/or Reference
WSA-3426	I	South Asia	Centers for Disease Control/CDC0382
WSA-3432	I	South Asia	Centers for Disease Control CDC0388/B8441
WSA-3533	I	South Asia	Suda Chaturvedi (19–36)
WSA-3534	I	South Asia	Suda Chaturvedi (19–37)
WSA-3425	II	East Asia	Centers for Disease Control/CDC0381
WSA-3427	III	Africa	Centers for Disease Control/CDC0383
WSA-3428	III	Africa	Centers for Disease Control/CDC0384
WSA-3543	III	Africa	Suda Chaturvedi (19–65)
WSA-3535	IV	South America	Suda Chaturvedi (19–62)

**Table 2:** Oligonucleotides and probes.

Primer or Probe	Sequence
B8441.Not.CEN1.F	5′-AAAGCGGCCGCTGGCGGGGGTCAATTTTCAG-3′
B8441.Sac.CEN1.R	5′-AAACCGCGGCAGTAAGCTTCTACGCACTGGA-3′
B8441.Not.CEN2.F	5′-AAAGCGGCCGCGCGGAAGGGCTAAACCTCTT-3′
B8441.Sac.CEN2.R	5′-AAACCGCGGATCGATGAGGTTGATCTTGAGCTT-3′
B8441.Not.CEN3.F	5′-AAAGCGGCCGCTTGATGCACCCAGAGGCAAA-3′
B8441.Sac.CEN3.R	5′-AAACCGCGGGAGTAGGGCAGAAGTTGATCTACAA-3′
B8441.Not.CEN 4.F	5′-AAAGCGGCCGCTTGGCATCTGCTTCCTTGCT-3′
B8441.Sac.CEN 4.R	5′-AAACCGCGGGCACATGCAATCGCTATCAAGTAG-3′
B8441.Not.CEN5.F	5′-AAACCGCGGTTTGGTGGAAAGGAAGGTGACA-3′
B8441.Sac.CEN5.R	5′-AAACCGCGGTTTGGTGGAAAGGAAGGTGACA-3′
B8441.Not.CEN6.F	5′-AAAGCGGCCGCGCTCTCAGCTCCGGTCTTTT-3′
B8441.Spe.CEN6.R	5′-AAAACTAGTTGAGAAGATACGGCCTACCCA-3′
B8441.Not.CEN7.F	5′-AAAGCGGCCGCCGGTGACAGGGAACTTCAAGT-3′
B8441.Sac.CEN7.R	5′-AAACCGCGGCCTCATGGCGGCCAATTACA-3′
CEN 4.NotI.R	5′-AAAGCGGCCGCTCTAGAACTA-3′
CEN 4.NotI.6188.F	5′-AAAGCGGCCGCCTTTGAAGTTGGCTGTGATGGA-3′
CEN 4.NotI.5622.F	5′-AAAGCGGCCGCCAAATTTCTGCAGACTGGTGTATC-3′
CEN 4.NotI.5200.F	5′-AAAGCGGCCGCCATACTTCACCGCCAAAGAAAGA-3′
CEN 4.NotI.4681.F	5′-AAAGCGGCCGCGCAATGATATACGGAGGAGTTGTG-3′
CEN 4.NotI.4199.F	5′-AAAGCGGCCGCGAAATGCACCATGCACGAAAC-3′
CEN 4.NotI.3684.F	5′-AAAGCGGCCGCTTCAGCTGCTTTCGCAGTATC-3′
CEN 4.SacII.R	5′-AAAGCATGTGCCCGCGGTGG-3′
CEN 4.SacII.4162.F	5′-AAACCGCGGAAGGTGAGGATTCGAAGGGAAGGA-3′
CEN 4.SacII.3621.F	5′-AAACCGCGGAGGAGGTTACGCGAACGACAAGTT-3′
CEN 4.SacII.2963.F	5′-AAACCGCGGAGCTAGAAGCACGGGTTCCAACAT-3′
CEN 4.SacII.2347.F	5′-AAACCGCGGTGAAGCAACGAATGGTGGCCATA-3′
CEN 4.SacII.1871.F	5′-AACCGCGGTGCCCGTAGACTTCCTGTCCATT-3′
CEN 4.SacII.1823.F	5′-AAACCGCGGACACCAGTCTGCAGAAATTTGCTTTAT-3′
CEN 4.SacII.1274.F	5′-AAACCGCGGATTCGACTACCGATCCATCACAGC-3′
CaurisADE1.5Kb.F	5′-AAAGCGGCCGCCCCATCTTGCAGCTAAGCGA-3′
CaurisADE1.5Kb.R	5′-AAAACTAGTAAACGAGGCTCATGACGAGG-3′
CaurisADE1.10Kb.F	5′-AAAGCGGCCGCCCTGGCTCGATCTTACAGGC-3′
CaurisADE1.10Kb.R	5′-AAAACTAGTAAAGCTCCCAACCTGTCACC-3′
C.aur.ACT.qPCR.F	5′-TTGGACTTTGAGCAGGAGATG-3′
C.aur.ACT.qPCR.R	5′-CTCTCGTTTCCGATGGTGATAA-3′
C.aur.NAT.qPCR.F	5′-CCAGTTGATCCACCATTGACTA-3′
C.aur.NAT.qPCR.F	5′-AACAACGAAACCAGCCAAATC-3′
Actin.Fam Probe	5′-/FAM/TCCTCTCAG/ZEN/TCGTCCGCT-3′
NAT.Cy5 Probe	5′-/Cy5/TCCGATGCT/TAO/GGTGAAGATG-3′

**Table 3:** Plasmids.

Plasmid Number	Plasmid Size	Fragment(s)	Description or Reference
p624	9.5 kb	NAT1	Nourseothricin resistance [28]
p1114	5 kb	NAT1	pBluescript SK(+) + NAT1
p1124	10.2 kb	CEN-1	p1114 + CEN 1 fragment
p1129	10 kb	CEN-2	p1114 + CEN 2 fragment
p1130	11.8 kb	CEN-3	p1114 + CEN 3 fragment
p1120	8.6 kb	CEN-4	p1114 + CEN 4 fragment
p1123	11 kb	CEN-5	p1114 + CEN 5 fragment
p1134	9.8 kb	CEN-6	p1114 + CEN 6 fragment
p1122	10.5 kb	CEN-7	p1114 + CEN 7 fragment
p1137	8.1 kb	p1114 + 6188 *NotI* (CEN 4)	*NotI* subclone of CEN 4
p1138	7.6 kb	p1114 + 5622 *NotI* (CEN 4)	*NotI* subclone of CEN 4
p1139	7.1 kb	p1114 + 5200 *NotI* (CEN 4)	*NotI* subclone of CEN 4
p1140	6.6 kb	p1114 + 4681 *NotI* (CEN 4)	*NotI* subclone of CEN 4
p1141	6.1 kb	p1114 + 4199 *NotI* (CEN 4)	*NotI* subclone of CEN 4
p1142	5.6 kb	p1114 + 3684 *NotI* (CEN 4)	*NotI* subclone of CEN 4
p1143	8.3 kb	p1114 + 4162 *SacII* (CEN 4)	*SacII* subclone of CEN 4
p1146	7.8 kb	p1114 + 3621 *SacII* (CEN 4)	*SacII* subclone of CEN 4
p1148	7.1 kb	p1114 + 2963 *SacII* (CEN 4)	*SacII* subclone of CEN 4
p1149	6.5 kb	p1114 + 2347 *SacII* (CEN 4)	*SacII* subclone of CEN 4
p1151	6.0 kb	p1114 + 1823 *SacII* (CEN 4)	*SacII* subclone of CEN 4
p1152	5.4 kb	p1114 + 1274 *SacII* (CEN 4)	*SacII* subclone of CEN 4
p1153	6.7 kb	p1114 + *NotI* 6188-*SacII* 2963	Minimal CEN 4 subclone
p1210	11.6 kb	p1153 + *NotI*-*SpeI ADE1* fragment	5.0 kb ADE1 PCR product digested with *NotI*-*SpeI* and cloned into p1153 digested with same
p1213	16.6 kb	p1153 + *NotI*-*SpeI ADE1* fragment	10.0 kb *ADE1* PCR product digested with *NotI-SpeI* and cloned into p1153 digested with same

## Data Availability

Raw data are contained within the article or Supplementary Materials. Plasmids were deposited with Addgene (see Section 2 for accession number).

## Supplementary Materials

The following supporting information can be downloaded at: https://www.mdpi.com/article/10.3390/jof10070477/s1, Figure S1: Poly (T) regions within CEN 4. Figure S2: Predicted fragment sizes and restriction maps of p1153 (6.6 Kb) digested with EcoRI, p1210 (11.6 Kb), digested with XbaI, and p1213 (16.6 Kb) digested with XbaI. Figure S3: qPCR raw data.
