# Supplementary material for: Development of a Shuttle Vector That Transforms at High Frequency for the Emerging Human Fungal Pathogen: Candida auris
Source: J Fungi (Basel). 2024 Jul 11;10(7):477. doi: 10.3390/jof10070477 (PMC11278357; doi:10.3390/jof10070477)
Supplement: Supplementary file 1 [file jof-10-00477-s001.zip › jof-3087130-Supplemental Figures.pdf]

GGCCGCTTGGCATCTGCTTCCTTGCTGCAAGATATTATTGGCATGGTAATTAACACGGAATGGGGTTCATTTGACAACCTGTTGGATGTGTTGCCAAGAACTAAATTTGATGATGTAGTAGATTCGGAAACATCAAAC
AAAGGCTATCATCTCTTTGAAAAGAGAATCAGCGGAATGTTCTTAGGTGAACTTTTAAGAGTGACACTCCTTGATCTCTTTGAACGCAAACTAGTATTCCAAGATCTTTATAAGAAGAGAGGGGGAAGTTTGCCCCAT
CGCTTAAGAGAGCCCTTTCTCTTGTCAGCTGAAGTGTTGTCGTATATCGAAATTGACGACTCAACTGAACTTAGGATGTCAGCTCTTATTCTCGAGAATCACTTGCGCCTTCCAACTACCTACGAGGAAAGAATTGCT

NotI.6188

ATCCAGAAACTCACACAGGCAATATCAAACAGGGCCGCGTGTCTTTCCGCTATACCTTTGGCTGCTATAGTTATGAGGGTCAAGGACCAATACGTCGATGATGATAAAGACTTTGAAGTTGGCTGTGATGGATCGGTA
GTCGAATTTTACCCGGGATTTCAAGAAAAGATTTTAAATGCATTTAATCTTATTAACCCGTTGAAGGGATCAAACAAGAAATTGCATTTGCAGATTGCGAAAGACGGTAGTGGTGTTGGAGCTGCTCTTTGTGCATCC
ACCGTGACTTGAAAAAATGAATACGAAGATAACCTTATTATAAATTTAGCAGGAACTGTAATTGACGACTATCGTAACTTTGCTAGTTTTCCTTTAATA**TTTTTTTTTTTTTTTTTTTTTTTTTTTTT**CTTTTTCCAG
GTCACTCGGTTATGACGGACTACGTATTAGTGCAACTTTAAGACCGATCATACCTGTGTTCAAGTAGATTTTCCTTCGAACAAAGTTTTTTTAATCAAATAGTATCTTATTCTGATAAAATGGTAGGCCGGAATAGAA
TCGATAAAAAATTGATTATTTGAAAAATTGATTATTTGTTAAAAATAGGAGAAGCATACACAAACTATACGAAAGTAAGCTTATGTTCATCTCATATGTTGATAAGCACTCTACTTCTATAAAGCAAATTTCTGCAGA
CTGGTGTATCATGCTGTCTCGAGCCGCCAATGGACAGGGAAGTCTACGGCACTTCAAAAGGTAATTTCGAGTCTTCCAATGGTCTTGCCTTCTCCCGGAAGACTCTATATTCTATTCATAATTATCAGGAGACAGAAA
AAATCCACTAAGAAAAGCAAACAAAAGAAGTTGGATTTTTAAGTTAACGGGGCTTGAGTGACGGTGAGATTAGATACCACGAAGTTCGAATGTGTGTCTCCAAAAAGGCATACGCTCGATCGTTTTTACCGCGATTGA
AAAGAGTTTGCAAAGAAAAGTTGGAATAGAGGAAGATTGTTGGTTACCGAAGATACCAACATGAATTGATTTCTAAATATTTGAGAAGATTGTTGGTATCTCCACACATTCATATCCACATGCACAAAGTTTCATACT
TCACCGCCAAAGAAAGAAAAGTAAAGAATACAAGTGTTTGATTATTAATCTATTGTCCTTGAAGGCATAACGTATGTCACTTAAGCAAAATATGGCCACCATTCGTTGCTTCAATCATATTTTGGTGTAAGCAAATTA
TCCCGTTCGATAGATCTGTAAAAAATAAGCGAGAAAACTACCAGTGTATGAAGCGATAGACGACATCAATGTATCCAAATGTTTTAAGAAAAAAGATTCAGGCAAAATATGATCTTTTCATTCTTGTATTGTAAGAAA
TGAATTCGTGACTTTTTCATATTTAATTTAAGTTAGTGACCCCTTGACAGTTTCATTTGCTTAAATCATTTGAAATGTTGCATACTCGTTTTTTTCGCAGTAGTCTTTTAATTAG**TTTTTTTTTTTTTTTTT**CTTTCA
TTACGCCTATGTAGCGAGTCAATCGGCTGAATGCGCAAACCTTGAACTCGATTTTATTATTTTTTAATCCAAGTACATACAACATAATTCCTGTCTAATGCAATGATATACGGAGGAGTTGTGCCTGGGTAGCGGTTC
GTATGCGAGGTTGCTTTGTTCACTGAATAGTTTTCATTGCCTCCATTTTATGATGTTATGTAGTTAAGTACACAAGTAGTAGAACGGATGAGTGGTTTAATACTTTTGATCCATATGATCTACGAGTATATTTTTCTT

SacII.2963

TAGCGAATGTGCTCCGATGTTGGAACCCGTGCTTCTAGCTAAACTTTAGTTTTTATTACTCAGTTGATCAAATTCGCTGCTGTTAAGCTGTCATTTTTAATGTGAAATTTTTACTGCCTTTGGGGACACCACCCTAGC
ATTGAGTAGTTTATTTTTCAATTACTGTTATGTGTTTCCTTGCTCTGCGATATATGTGATAGCAATGAAATGATAAATTCTAGCATACCTTTATTTTATGAATCTTTAAATCATACGAAATGAGCTTGAAGCTCCTAT
CTCATTTCTATGCATTTCCAAAAACCTCAGAAATGCACCATGCACGAAACACATACCATTGCTAACTTAGTCAAGGTTTTATATTTTGTCTGCAATTTTGATGTTTATTCATTTTAGAAGTTCAGCAAGTTGAATGAT
CATATTGTTTTATGTGTGCAGCATGCAAATTGGAAGTCATTCGTAAAGAGATTAGAGAAAATTACTTAATGGACTCGATGGCTACGAACTGTGTAGACATGTATTATATTTGAAATTTATCTGAATATTTCAGTGGTA
TAAGTTGAGAATCTGATGATATTCATTCTTCATCGACCAAACTGAATCTGATTATAAGCCCAATGTGACCTTTTTTAGCGCCATCTAGCCCGAATTCCTTAAGAACCCAGTTGAAGCTGTTCAACTTGTCGTTCGCGT
AACCTCCTACGTCTATGTCAAGCGACTTAGTCCCATAAGTAGGAATCAGTAGCTTATATTGAGAATTAGCCAATTTGACATAATTGGTGTCGAACTGAAAAGTGTAATCGTGGTCTTGATCAAAAATGAATTCAGCTG
CTTTCGCAGTATCGGAAAAATACTTGCTTCTTGCGGTGCAGTGATCTGGAATGTCTAAAGAGCTAACGTCTCCTAAGTTCTCTGTATCATCATCTAGGGGGTATAGTTTGTCACCCTTTTGCATACAATGATCTTCAG
CATAAATCTTAGTAAAGCTATTCAAAGCGAGACCGAATAAAAAAGGCATATTGTTATACAAATCACCCTTCACAGAAGGGTTTAACCACCACGAGAAAAACTTCAAGCCAGTCGACAATAGGGAGACAGGGACGTAAT
CTTTGATCGGTGTAAGGGATTCATTTCCGAATACAAGATCGGACCCATTCATTGGTCTTGCCGGATTAAATTTAATGAGAATTCTTAAATTGGGCAATGTATCTGCTTGGATCCTTCCCTTCGAATCCTCACCTTCAC
TTGAAGCGGCGTCATCAGCGAAGTTGTATTTACTGTTGTCTCGGTGAGGTTCACTTCCTTCGAAATCCCTAATGAAAATTTGAAGCTGACATACCCCGATGTCCGACTGTAATACAATGGCTTCAGTAGAATTCACTG
GAACTGTTAAAAATTGAGCTTCGTACGAGGAGGAAGCTTCAATGAGAAGCCGTTTGACAACCATCAAGAAAAGAACTGCTTTAGTTTAGAACAACCGCTAGGTTGCGCAACAATATAAAGAATTTAGGTTATCCACAA
TCCTTGCTACTTGATAGCGATTGCATGTGCCCGC

Supplemental Figure S1. Poly (T) regions within CEN 4. *Candida auris* centromeres have long stretches of poly (T) regions within or close to the core centromere. Two such regions were identified within the minimal functional CEN 4 subclone. Exclusion of these regions from subclones severely reduces transformation frequency and colony size. Subclone regions are shown in blue lettering with the individual subclone restriction site shown directly above, in bold lettering. Poly (T) regions are shown in red lettering.

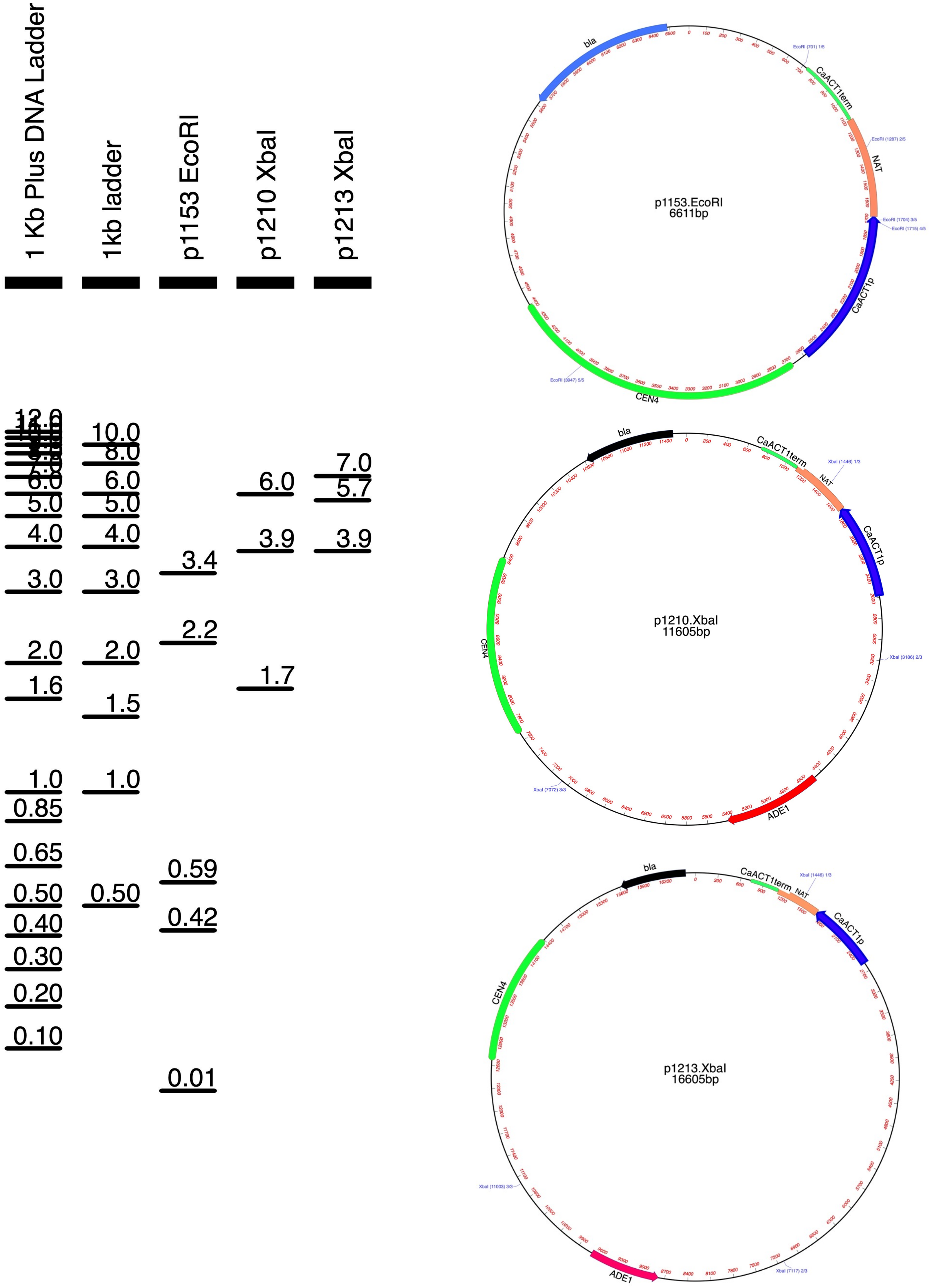

Supplemental Figure S2. Predicted fragment sizes and restriction maps of p1153 (6.6 Kb) digested with EcoRI, p1210 (11.6 Kb), digested with XbaI, and p1213 (16.6 Kb) digested with XbaI.

Supplemental Figure S3. *qPCR raw data.*

	Small Colony	NAT Cq	ACT Cq	Small colony- Copy #	Large Colonies	NAT Cq	ACT Cq	Large Colony - Copy #
Expt 1	Col 1	24.92	24.32	0.66	Col 1	23.13	23.3	1.13
	Col 2	25.29	24.92	0.77	Col 2	22.07	22.32	1.19
	Col 3	25.45	25.33	0.92	Col 3	21.22	21.56	1.27
	Col 4	22.9	20.53	0.19	Col 4	18.41	18.69	1.21
	Col 5	20.06	20.28	1.16	Col 5	19.47	20.03	1.47
	Col 6	25.89	20.51	0.02	Col 6	20.16	20.52	1.28
	Col 7	20.06	20.69	1.55	Col 7	20.21	20.67	1.38
Expt 2	Col 1	24.35	24.73	1.30	Col 1	24.65	25.67	2.03
	Col 2	24.73	25.21	1.39	Col 2	22.01	23.08	2.10
	Col 3	24.57	25.40	1.77	Col 3	20.61	21.76	2.22
	Col 4	21.84	22.33	1.40	Col 4	17.87	19.17	2.46
	Col 5	18.98	20.54	2.94	Col 5	19.08	20.3	2.33
	Col 6	24.67	24.57	0.93	Col 6	20.06	21.11	2.07
	Col 7	19.27	20.84	2.97	Col 7	19.99	21.36	2.58
Expt 3	Col 1	24.78	24.9	1.09	Col 1	24.57	25.52	1.93
	Col 2	25.28	25.56	1.21	Col 2	22.25	23.18	1.91
	Col 3	25.08	25.7	1.54	Col 3	20.55	21.74	2.28
	Col 4	22.49	22.56	1.05	Col 4	17.68	19.01	2.51
	Col 5	19.41	20.72	2.48	Col 5	18.85	20.14	2.45
	Col 6	25.5	24.96	0.69	Col 6	19.79	21.03	2.36
	Col 7	20.01	21.15	2.20	Col 7	20.51	21.84	2.51
Expt 4	Col 1	26.68	26.34	0.79	Col 1	20.91	21.89	1.97
	Col 2	24.05	24.02	0.98	Col 2	18.8	20.25	2.73
	Col 3	24.13	24.58	1.37	Col 3	19.69	20.75	2.08
	Col 4	24.17	24.84	1.59	Col 4	17.17	18.15	1.97
	Col 5	21.64	21.76	1.09	Col 5	18.09	19.18	2.13
	Col 6	24.78	24.25	0.69	Col 6	19.24	20.4	2.23
	Col 7	19.25	20.37	2.17	Col 7	19.15	20.34	2.28
Expt 5	Col 1	26.77	26.29	0.72	Col 1	20.97	21.95	1.97
	Col 2	23.8	23.81	1.01	Col 2	19.25	20.48	2.35
	Col 3	24.06	24.42	1.28	Col 3	20.08	20.94	1.82
	Col 4	24.17	24.69	1.43	Col 4	17.2	18.33	2.19
	Col 5	21.73	21.93	1.15	Col 5	18.35	19.23	1.84
	Col 6	24.71	24.14	0.67	Col 6	19.32	20.43	2.16
	Col 7	19.2	20.25	2.07	Col 7	19.35	20.52	2.25
**Copy Number Average**				**1.29**				**2.02**

Small Colonies NAT Cq	
Mean	23.27622581
Standard Error	0.402849424
Median	24.17
Mode	24.78
Standard Deviatio	2.383289332
Sample Variance	5.680068039
Kurtosis	-0.931823449
Skewness	-0.639628426
Range	7.79
Minimum	18.98
Maximum	26.77
Sum	814.6679032
Count	35

Large Colony NAT Cq	
Mean	20.02028571
Standard Error	0.305954488
Median	19.79
Mode	#N/A
Standard Deviation	1.810051162
Sample Variance	3.27628521
Kurtosis	0.83202893
Skewness	0.861355979
Range	7.48
Minimum	17.17
Maximum	24.65
Sum	700.71
Count	35

Small Colonies ACT Cq	
Mean	23.35527704
Standard Error	0.347020184
Median	24.25
Mode	#N/A
Standard Deviatio	2.052999098
Sample Variance	4.214805295
Kurtosis	-1.470402727
Skewness	-0.363020696
Range	6.09
Minimum	20.25
Maximum	26.34
Sum	817.4346964
Count	35

Large Colony ACT Cq	
Mean	20.99542857
Standard Error	0.29195497
Median	20.67
Mode	20.52
Standard Deviation	1.727228897
Sample Variance	2.983319664
Kurtosis	1.314718087
Skewness	0.902765255
Range	7.52
Minimum	18.15
Maximum	25.67
Sum	734.84
Count	35

t-Test: Two-Sample Assuming Equal Variances

	Small - Copy Number	*Large - Copy Number*
Mean	1.293653361	2.01864077
Variance	0.455006568	0.18894987
Observations	35	35
Pooled Variance	0.321978219	
Hypothesized Mean Differenc	0	
df	68	
t Stat	-5.344858862	
P(T<=t) one-tail	5.68687E-07	
t Critical one-tail	1.667572281	
P(T<=t) two-tail	1.13737E-06	
t Critical two-tail	1.995468931	
